# Effects of *Artemisia annua* supplementation on the performance and gut health of laying hens challenged with mixed *Eimeria* species

**DOI:** 10.3389/fphys.2024.1381548

**Published:** 2024-05-15

**Authors:** Milan Kumar Sharma, Guanchen Liu, Venkata Sesha Reddy Choppa, Hamid Reza Rafieian-Naeini, Fatemeh Sadat Mahdavi, Brett Marshall, Robert M. Gogal, Woo Kyun Kim

**Affiliations:** ^1^ Department of Poultry Science, College of Agricultural and Environmental Sciences, University of Georgia, Athens, GA, United States; ^2^ Department of Biosciences and Diagnostic Imaging, College of Veterinary Medicine, University of Georgia, Athens, GA, United States

**Keywords:** *Artemisia annua*, phytogenic feed additive, laying hens, coccidiosis, gut health, egg production

## Abstract

**Background:**

Coccidiosis outbreaks in susceptible laying hens can significantly decrease egg production and cause substantial economic loss to the egg industry. The supplementation of poultry diets with chemotherapeutic agents is limited due to antimicrobial resistance and residue in poultry meat or processed products. Therefore, alternative strategies to control coccidiosis are needed, and *Artemisia annua* (**AA**) might have the potential to be a phytogenic feed additive, an alternative to anticoccidial agents. This study aimed to investigate the effect of the dietary supplementation of powdered AA on the performance and gut health of laying hens infected with coccidiosis by *Eimeria* spp.

**Methods:**

A total of 225 Hy-Line W-36 laying hens at 23 weeks of age were allocated into 5 treatment groups: 1) control (**NC**), 2) pair-fed (**PF**) control, 3) challenged control (**CC**), 4) CC with dietary inclusion of 0.5% AA (**0.5AA**), and v) CC with dietary inclusion of 1% AA (**1AA**). The hens in the CC, 0.5AA, and 1AA groups were orally inoculated with sporulated oocysts of *Eimeria maxima* (12,500), *Eimeria tenella* (12,500), and *Eimeria acervulina* (62,500) at week 25. The PF hens received the same amount of feed consumed by the CC hens from 0–14 days post-inoculation (**dpi**) of *Eimeria* spp. The performance of the laying hens, including body weight (**BW**), hen–day egg production (**HDEP**), feed intake (**FI**), and feed conversion ratio (**FCR**), was measured weekly. Additionally, markers of intestinal health, including gut permeability, lesion score, intestinal morphometry, and immune responses, were evaluated at 6, 14, and 21 dpi.

**Results:**

At 6 and 14 dpi, laying hens challenged with *Eimeria* spp. had a lower BW than PF and NC hens (*p <* 0.0001). Supplementation of 1% AA improved the HDEP by 8.1% compared to CC hens; however, it was still 15.4% lower than that of PF hens (*p <* 0.0001). The inclusion of 1% AA did not have any beneficial effect on FI; however, the FCR was improved by 0.61 (2.46) than that of CC hens (3.07; *p <* 0.0001). The inclusion of 1% AA reduced the severity of the intestinal lesions and increased the recovery of intestinal villi (*p <* 0.05). Additionally, gut permeability was significantly different between the challenged and non-challenged hens; however, among the challenged hens, the inclusion of AA reduced the gut permeability by 29% compared to CC hens (*p* < 0.0001). Furthermore, the inclusion of 0.5% AA reduced the inflammatory responses in the infected hens.

**Conclusion:**

Dietary inclusion of AA partially restored the performance and gut health of the laying hens and modulated their inflammatory immune response following *Eimeria* infection; however, further studies are needed to better understand the mode of action and effective dosages to improve the gut health without negative impacts on the performance.

## Introduction

The poultry industry worldwide is under tremendous pressure to control and minimize the risk associated with avian coccidiosis (Abbas et al., 2011). Despite several strategies (biosecurity, vaccination, or the use of coccidiostat or coccidiocidal drugs) being employed to control coccidiosis in commercial poultry production, coccidiosis is still the leading cause of economic loss in the poultry industry (Peek and Landman, 2011; Liu et al., 2023). Unfortunately, the development of resistance against currently available coccidiostat or coccidiocidal drugs, potential drug residues in the final products, and consumer interests have challenged the industry for alternatives to control coccidiosis. Recently, the use of phytogenic feed additives has been increasing as it has been shown to improve the performance, immune response, and intestinal barrier functions against coccidiosis (Choi and Kim, 2020; Yadav et al., 2020; de Andrade et al., 2022; El-Shall et al., 2022; Paneru et al., 2022; 2023).

*Artemisia annua* (AA), also known as sweet wormwood, is an herb used in traditional Chinese medicine to treat malaria caused by multidrug-resistant *Plasmodium* spp (Klayman, 1985). Artemisinin, a bioactive flavonoid found in AA leaves, inhibits the growth of several stages of *Plasmodium* spp. (Meshnick, 2002; Golenser et al., 2006). Like *Plasmodium* spp., *Eimeria* spp. is an intracellular parasite belonging to the same family, Apicomplexa. The use of AA and its extract, artemisinin, exhibit anticoccidial effects against *Eimeria tenella* (Allen et al., 1997; Del Cacho et al., 2010; Hady and Zaki, 2012; Drǎgan et al., 2014; Jiao et al., 2018) when infected by it alone. However, in the field, infection from only one species is rare. The use of either dried AA leaves or its extract, artemisinin, has shown potential in improving lesion scores, reducing oocyst shedding and sporulation, and modulating the humoral and immune response in chickens (Allen et al., 1997; Arab et al., 2012; Gholamrezaie et al., 2013; Kaboutari et al., 2014; Wan et al., 2016; Song et al., 2017; Yang et al., 2021). It has been observed that apicomplexan parasites have developed an anti-apoptotic mechanism to survive intracellularly in the host (Jiao et al., 2018). During coccidia infection, *Eimeria* alters the host cellular pathways, inhibiting the apoptosis of infected cells by upregulating the transcriptional factor (**NF-κB**) and the anti-apoptotic factor in chickens as a defense mechanism. However, artemisinin suppresses the expression of NF-κB and promotes the apoptotic pathways, limiting the *Eimeria* infection and inflammation (Jiao et al., 2018). Furthermore, artemisinin from AA alters the cell wall formation of the oocysts by disrupting the Ca^2+^ homeostasis of the oocysts, leading to the death of the developing oocysts and a reduction in the sporulation rate (Del Cacho et al., 2010). Artemisinin disrupts Ca^2+^ homeostasis in developing oocysts, affecting Ca-dependent pathways involved in protein secretion and differentiation. This disruption inhibits cell wall formation and leads to the degeneration of micro/macrogametes, ultimately reducing sporulation rates and oocyst shedding (Del Cacho et al., 2010). Therefore, this study aimed to evaluate the effect of dried AA leaves on the performance, gut health, and immune response of laying hens infected with mixed *Eimeria* species.

## Materials and methods

### Laying hen husbandry and experimental design

The Institutional Animal Care and Use Committee of the University of Georgia approved the animal study protocol (A2021 12–012). A total of 225 23-week-old Hy-Line W-36 laying hens were randomly divided into 5 treatment groups (n = 45), each comprising 5 replicates. The laying hens were adapted to the diets for 2 weeks. At 25 weeks of age, the hens were challenged with mixed *Eimeria* species (12,500 *Eimeria maxima*, 12,500 *E. tenella*, and 62,500 *Eimeria acervulina* oocysts) according to the treatment groups. The five different treatments were 1) non-challenged and fed a basal diet (**NC**), 2) non-challenged and pair-fed (**PF**), 3) basal diet with mixed *Eimeria* spp. challenge (**CC**), 4) dietary inclusion of 0.5% AA leaf powder and challenged with mixed *Eimeria* spp. (**0.5AA**), and 5) dietary inclusion of 1% AA leaf powder and challenged with mixed *Eimeria* spp. (**1AA**). The powdered AA leaves were purchased from Artennua^®^ (Barcelona, Spain). The experimental mash diets were isocaloric and isonitrogenous and formulated to meet the nutrient requirements of laying hens (Hy-Line International, Mansfield, GA). The nutritional composition of AA powder and diets are shown in Tables 1, 2, respectively. Feed and water were provided a*d libitum* throughout the experimental period except for the PF group. The PF group was fed the exact amount of the basal diet consumed by the CC group from 0–14 days post-inoculation (dpi) of *Eimeria* spp. (**dpi**). The feed consumption of the CC group was measured daily at the same time following the *Eimeria* challenge, and the average daily feed consumption was calculated. The exact amount of feed was then provided to the PF group on the next day. To ensure that the PF group had the same amount of feed as the CC group, feed addition and consumption were carefully monitored to prevent any wastage. The laying hens were housed in environmentally controlled battery cages (475 cm^2^/hen) equipped with trough feeders and nipple drinkers. The lighting regime was adjusted based on the breeder management guidelines (Hy-Line International, Mansfield, GA).

**TABLE 1:** Nutritional composition and artemisinin content of dried *Artemisia annua* leaves.

Item^a^	Value	Unit
Gross energy	3,080	kcal/kg
Crude protein	9.80	%
Crude fiber	7.30	%
Ether extract	4.30	%
Total lysine	0.47	%
Total methionine	0.13	%
Total arginine	0.37	%
Total leucine	0.62	%
Total isoleucine	0.39	%
Total valine	0.46	%
Total threonine	0.34	%
Total cysteine	0.11	%
Total phenylalanine	0.41	%
Total tryptophan	<0.02	%
Total artemisinin	0.93	%

^a^ The samples were sent to the University of Missouri agricultural experiment station chemical laboratories for the above-mentioned analysis.

**TABLE 2:** Diet formulation and nutritional composition of experimental diets.

Ingredients (%)	Control	0.5AA	1AA
Corn	58.55	58.03	57.55
Soybean meal	22.00	22.00	22.00
Limestone^a^	9.34	9.34	9.34
Soybean oil	3.00	3.00	3.00
Distiller’s dried grains with soluble	2.50	2.50	2.50
*Artemisia Annua*	0.00	0.50	1.00
Glycine	1.74	1.68	1.68
Dicalcium phosphate	1.70	1.70	1.71
Salt	0.39	0.39	0.39
DL-methionine	0.33	0.40	0.39
Vitamin premix^b^	0.10	0.10	0.10
L-lysine	0.10	0.10	0.11
L-valine	0.09	0.09	0.10
L-threonine	0.08	0.09	0.10
Mineral mix^c^	0.075	0.075	0.075
**Calculated nutritional composition**			
ME (kcal/kg)	2910	2910	2910
Crude protein (%)	16.70	16.69	16.69
Total calcium (%)	4.00	4.00	4.00
Available P (%)	0.45	0.45	0.45
dLys (%)	0.80	0.80	0.80
dMet (%)	0.54	0.54	0.57
dTSAA (%)	0.73	0.73	0.76
dThr (%)	0.56	0.56	0.56
dTrp	0.16	0.16	0.16
dArg	0.89	0.89	0.89
dVal	0.71	0.70	0.70
dILeu	0.59	0.59	0.59

^a^ The level of the fine and coarse limestone ratio was maintained at 50:50.

^b^ Vitamin mix provided the following in mg/100 g diet: folic acid, 7.5; riboflavin, 1.5; nicotinic acid amide, 15; vitamin B-12 (source concentration, 0.1%) pyridoxine-HCl, 1.2; d-biotin, 3; thiamine-HCl, 1.5; 2; d-calcium pantothenate 4; menadione sodium bisulfite, 1.98; cholecalciferol (5,000,000 IU/g), 0.09; α-tocopherol acetate (500,000 IU/g), 22.8; retinyl palmitate (500,000 IU/g), 2.8; ethoxyquin, 13.34; dextrose, 762.2; and I-inositol, 2.5.

^c^ Mineral mix provided the following in g/100 g diet: CaCO_3_, 1.48; Ca(H_2_PO_4_)_2_ H_2_O, 3.62; Na_2_SeO_4_, 0.0002; KH_2_PO_4_, 1.00; FeSO_4_ 7H_2_O, 0.05; MnSO_4_ H_2_O, 0.035; KIO_3_, 0.001; MgSO_4_ 7H_2_O, 0.62; CuSO_4_·5H_2_O, 0.008; NaCl, 0.60; ZnCO_3_, 0.015; 0.0011; KCl, 0.10; and NaMoO_4_·2H_2_O, CoCl_2_ 6H_2_O, 0.00032.

### Laying hen performance

The laying hens were weighed at 23 and 25 weeks of age and 6, 14, and 21 dpi for growth performance. The number of eggs laid per replicate was recorded daily, and hen–day egg production (**HDEP**) was calculated. The daily feed consumption (**ADFI**) and feed conversion ratio (**FCR**) were measured. The FCR was expressed as the kg of feed consumed per dozen eggs produced (Sharma et al., 2022b).

### Gut permeability, lesion score, and oocyst shedding

At 5 dpi, 2.2 mg/mL of FITC-d (MW 4000; Sigma-Aldrich, Canada) was orally inoculated to one hen per replicate, and blood was collected 2 hours post-inoculation from the wing vein. The concentration of FITC-d that leaked into the bloodstream was measured using a plate reader at an excitation/emission wavelength of 485/525 nm (SpectraMax ABS Plus, Molecular Devices, San Jose, CA). At 6 dpi, 2 hens per replicate were euthanized, and intestinal lesion scoring was carried out for *E. acervulina* (in the duodenum), *E. maxima* (in the jejunum and ileum), and *E. tenella* (in the ceca) on a scale of 0–4 following the methods described by Johnson and Reid, (1970). In brief, the lesion scores were ranked based on the severity of lesions from 0 to 4, with 0 indicating no lesions and 4 indicating extremely severe lesions. The severity of intestinal lesions was determined based on the density of the characteristic lesion of each *Eimeria* species, inflammation and thickening of the intestinal wall, and intestinal content at their predilection sites (Johnson and Reid, 1970). On 5, 6, 7, and 8 dpi, feces were allowed to accumulate on a manure belt. On day 8, the feces on the belt were thoroughly mixed within each replicate, and feces samples were collected for oocyst shedding. The oocyst counts for *E. acervulina*, *E. maxima*, and *E. tenella* were counted following the procedure proposed by Goo et al. (2023). In brief, 5 g of the feces was mixed in 35 mL of tap water and left to soften for 48 h at room temperature. Afterward, the sample was vortexed and diluted in a saturated saltwater solution (1:11 ratio). Then, 650 µL of the diluted solution was added to a McMaster counting chamber (Vetlab Supply, Palmetto Bay, FL) for oocyst enumeration. The different species were identified under a microscope based on the size and morphology of the oocysts.

### Sample collection and analysis

At 6, 14, and 21 dpi, 1 hen per replicate was randomly selected for sampling and was euthanized by cervical dislocation. For intestinal morphometric analysis, 2-cm-long sections of the duodenum, jejunum, and ileum were collected and fixed in 10% neutral-buffered formalin. The villus height (**VH**), crypt depth (**CD**), and VH-to-CD (**VHCD**) ratio were measured following the procedure outlined by Sharma et al. (2024b). Furthermore, samples of the jejunum and cecal tonsils were collected and snap-frozen in liquid nitrogen at the abovementioned time points for gene expression. The RNA extraction from the above samples was carried out using the TRIzol reagent (QIAGEN; Valencia, CA) following the procedures proposed by Sharma et al. (2022a). In brief, the total RNA concentration was determined using the NanoDrop-8 spectrophotometer (Thermo Fisher Scientific, Waltham, MA). It was later reverse-transcribed to cDNA after normalizing it to 2 μg/μL using a commercial kit (Applied Biosystems, Foster City, CA), and qRT-PCR was performed in duplicate. The ΔCt values of each marker gene from the NC group were used to calculate the ΔΔCt value. The expression levels of the NC group were then normalized to 1 using the 2^−ΔΔCt^ method, and the relative fold change of the mRNA expression levels was calculated using the 2^−ΔΔCT^ relative quantification methods compared to the NC group. The list of primers for the target and housekeeping genes is shown in Table 3.

**TABLE 3:** List of the nucleotide sequences of the housekeeping and target genes used in this study.

Gene^a^	Accession number	Nucleotide sequence
Housekeeping gene		
ACTB	NM_205518.2	F: CAACACAGTGCTGTCTGGTGGTA R: ATCGTACTCCTGCTTGCTGATCC
Intestinal barrier functions		
JAM-2	XM_025149444.1	F: AGCCTCAAATGGGATTGGATT R: CATCAACTTGCATTCGCTTCA
ZO-2	XM_025144669.1	F: GGCAAATCATTGAGCAGGA R: ATTGATGGTGGCTGTAAAGAG
CLDN-1	NM_001013611.2	F: TGGAGGATGACCAGGTGAAGA R: CGAGCCACTCTGTTGCCATA
OCLN	NM_205128.1	F: ACGGCAGCACCTACCTCAA R: GGCGAAGAAGCAGATGAG
MUC-2	NM_001318434.1	F: ATGCGATGTTAACACAGGACTC R: GTGGAGCACAGCAGACTTTG
iNOS	NM_204961.2	F: GAGCACTCATGACCCCAAAG R: GGGCCAGGTGCTCTTCTATT
Nutrient transporter genes		
b^0,+^ AT	NM_001199133.2	F: TTATCACCGCACCTGAAC R: AGCATCTGAAGGTGCATAG
*b* ^*0*^ *AT*	XM_040663289.2	F: GGGTTTTGTGTTGGCTTAGGAA R: TCCATGGCTCTGGCAGAGATTT
*EAAT-3*	XM_046936555.1	F: TGCTGCTTTGGATTCCAGTGT R: AGCAATGACTGTAGTGCA GAAGTAATATATG
*pepT-1*	NM_204365.2	F: CCCCTGAGGAGGATCACTGTT R: CAAAAGAGCAGCAGCAACGA
*y* ^*+*^ *LAT-1*	XM_046911929.1	F: AATGTGAAGTGGGGAACTCG R: CACCCTGCGTAGGAGAAGAG
SGLT-1	XM_046928028.1	F: GCCATGGCCAGGGCTTA R: CAATAACCTGATCTGTGCACCAGTA
*GLUT-1*	NM_205209.2	F: TCCTCCTGATCAACCGCAAT R: TGTGCCCCGGAGCTTCT
Inflammatory cytokines		
*IL-1β*	NM_204524.2	F: TGCCTGCAGAAGAAGCCTCG R: GACGGGCTCAAAAACCTCCT
*IL-4*	NM_001398460.1	F: CTTATGCAAAGCCTCCACAA R: TGGTGGAAGAAGGTACGTAGG
*IL-10*	NM_001004414.4	F: AGCAGATCAAGGAGACGTTC R: ATCAGCAGGTACTCCTCGAT
*IL-17*	NM_204460.2	F: TATCAGCAAACGCTCACTGG R: AGTTCACGCACCTGGAATG
*TNF-α*	MF000729.1	F: CGTGGTTCGAGTCGCTGTAT R: CCGTGCAGGTCGAGGTACT
*IFN-*γ	NM_205149.2	F: CACATATCTGAGGAGCTCTATAC F: GTTCATTCGCGGCTTTG

^a^ ACTB, beta-actin; JAM-2, junctional adhesion molecule-2; ZO-2, zonula occludin-2; CLDN-1, claudin-1; OCLN, occludin; MUC-2, mucin-2; iNOS, inducible nitric oxide synthase; b0,+ AT (SLCA9), solute carrier family 7, member 9; b0 AT (SC6A19), solute carrier family 6, member 19; EAAT-3 (SLC1A1), excitatory amino acid transporter 3; pepT-1(SLC15A1); y^+^ LAT-1 (SLC7A7), y^+^ L amino acid transporter-1; SGLT-1 (SLC5A1), sodium-glucose transporter-1; GLUT-1 (SLC2A1), glucose transporter-1; VDR, vitamin D receptor; IL-1β, interleukin-1 β; IL-4, interleukin-4; IL-10, interleukin-10; IL-17, interleukin-17 A; TNF-α, tumor necrosis factor-α; IFN-γ, interferon-γ.

The oxidative status of the laying hens following the *Eimeria* challenge was measured from 1 hen per replicate at 6, 14, and 21 dpi. The concentration of the total antioxidant capacity (**TAC**) in the serum was measured following the manufacturer’s instructions (BioAssay Systems, Hayward, CA). In brief, blood was collected from the wing vein, serum was separated by centrifugation at 1,000 × *g* for 15 min, and the TAC concentration was measured using a plate reader (λ = 485 nm; SpectraMax ABS Plus, SoftMax Pro 7 software, Molecular Devices, San Jose, CA). The superoxide dismutase (**SOD**) and malondialdehyde (**MDA**) concentrations in the liver were analyzed as a marker of oxidative status using commercial kits (MDA: BioAssay Systems, Hayward, CA; SOD: Cayman Chemical assay kits, Ann Arbor, MI). Approximately 50–70 mg of the liver sample was homogenized in the respective solutions following the manufacturer’s instructions, and the concentrations of MDA (λex/em = 530/550 nm) and SOD (λ = 450 nm) were measured using a plate reader (SpectraMax ABS Plus, SoftMax Pro 7 software, Molecular Devices, San Jose, CA). The concentrations of SOD and MDA were standardized for the protein concentration in the sample.

### Flow cytometry

**FIGURE 1:**
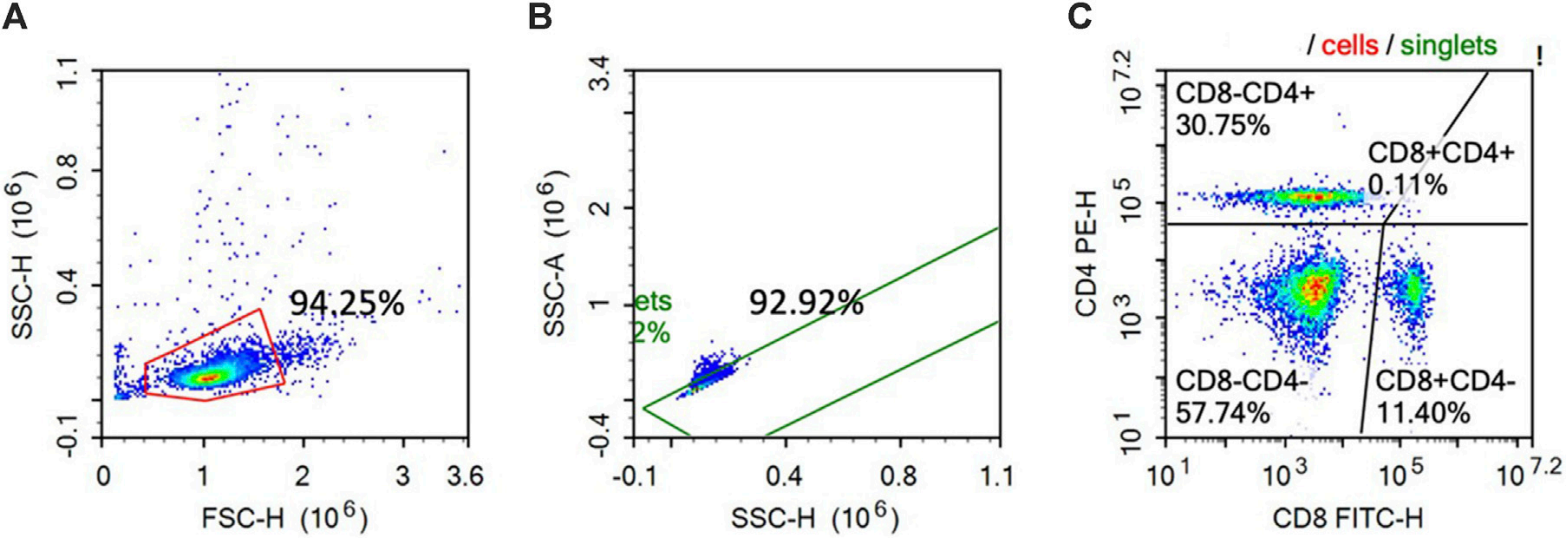
Representative histogram of the gating protocol used on the enriched chicken peripheral blood lymphocytes stained with PE-CD4 and FITC CD-8. The cells were first gated based on the size (FSC-H) and granularity (SSC-H) **(A)**. The single cell population was then gated from the histogram **(A)** based on the cell area (SSC-A) and the cell height (SSC-H) (Fig. 1B). Singlet cells identified in **(B)** were then analyzed for PE-CD4 and FITC-CD8 staining **(C)**.

At 14 dpi, 10 mL of blood was withdrawn from 1 hen per replicate through the wing vein into the Na-heparin glass tubes. The peripheral mononuclear blood cells were isolated following the procedures proposed by Choi et al. (2022) to a final concentration of 4.0 × 10^6^/mL. The CD4^+^ and CD8^+^ cell percentages were analyzed using flow cytometry following the procedures proposed by Krunkosky et al. (2020). In brief, 100 µL of the isolated cells were incubated with CD4 (mouse anti-chicken CD4-PE; SouthernBiotech, Birmingham, AL) and CD8 (mouse anti-chicken CD8a-FITC; SouthernBiotech, Birmingham, AL) antibodies. After incubation, the cells were washed with FACS-PBS and centrifuged at 250 *g* for 10 min, and the supernatant was discarded and resuspended in 100 μL FACS-PBS and 100 μL flow fixing solution (Thermo Fisher Scientific). Flow cytometry was conducted on a BD Accuri C6 Flow Cytometer (San Jose, CA). A total of 10,000 events were conducted per sample within the lymphocyte gate, and the expression percentages for each of the utilized markers were analyzed, as shown in Figure 1.

## Statistics

The data related to laying hen performance, gene expression, oxidative status, and intestinal morphology were analyzed using one-way analysis of variance in SAS (SAS Institute, Cary, NC). The lesion score data were analyzed as nonparametric data using the Kruskal–Wallis test. In contrast, the feed intake and egg production data were analyzed as split plots over time using the PROC GLM procedure of SAS (SAS Institute, Cary, NC). Statistical significance was set at *p* < 0.05, and the means were compared using Fisher’s LSD.

## Results

### Laying hen performance

The effects of feeding AA leaves on the growth performance of the laying hens following the *Eimeria* challenge are shown in Table 4. During the adaptation period, the BW of the hens fed 0.5% AA was reduced compared to the other treatment groups (*p* = 0.0642). At 6 dpi, the BW of hens infected with *Eimeria* spp. was reduced by at least 7% compared to 3% in the PF hens (*p* < 0.0001). However, at 14 dpi, the BW loss increased up to 8% in PF hens and at least 9% in the infected hens (*p* < 0.0001). The lower BW observed in the 0.5AA group during the adaptation period was exacerbated by the *Eimeria* infection, with the lowest BW observed at 14 dpi (*p* < 0.0001). By 21 dpi, the BW of the PF hens and challenged hens had recovered to levels comparable to those of NC hens (*p* = 0.1926).

**TABLE 4:** Effects of the dietary inclusion of phytogenic feed additive *Artemisia annua* in laying hen’s diets on the growth performance of the laying hens pre- and post-*Eimeria* challenge.

Items^a^	BW (initial)	BW (0 dpi)	BW (6 dpi)	BWG (0–6 dpi)	BW (14 dpi)	BWG (0–14 dpi)	BW (21 dpi)	BWG (0–21 dpi)
NC	1,571.5	1,568.9^a^	1544.7^a^	−24.17^a^	1,573.9^a^	5.07^a^	1,562.4	−6.48
PF	1,574.1	1,566.5^a^	1495.1^b^	−71.40^b^	1,448.6^b^	−117.93^b^	1,572.6	6.14
CC	1,569.3	1,571.7^a^	1,433.4^c^	−138.34^c^	1,422.7^bc^	−149.01^b^	1,566.4	−5.38
0.5AA	1,574.1	1,539.3^b^	1,401.2^d^	−138.05^c^	1,379.3^c^	−159.96^b^	1,521.6	−17.71
1AA	1,571.6	1,552.0^ab^	1,436.8^c^	−115.13^c^	1,408.7^bc^	−143.26^b^	1,524.5	−17.71
SEM	3.77	8.43	10.21	11.42	19.98	20.58	18.83	14.78
*p*-value	0.8776	0.0642	<0.0001	<0.0001	<0.0001	<0.0001	0.1926	0.8424

^a-c^values within columns not sharing the same superscripts are significantly different at *p* < 0.05.

^a^ BW, average body weight (g); BWG, average body weight gain (g/hen). At 6, 14, and 21 dpi, the number of laying hens per replicate was as follows: n = 9, n = 6, and n = 5, respectively. NC, non-challenged control; PF, pair-fed control; CC: challenged control (12,500 *E. maxima*; 12,500 *E. tenella*; and 62,500 *E. acervulina* oocysts per mL); 0.5AA, challenged control with the inclusion of 0.5% *Artemisia annua* in the diet; 1AA, challenged control with the inclusion of 1% *A. annua* in the diet; dpi, days post-inoculation of *Eimeria* spp.

**FIGURE 2:**
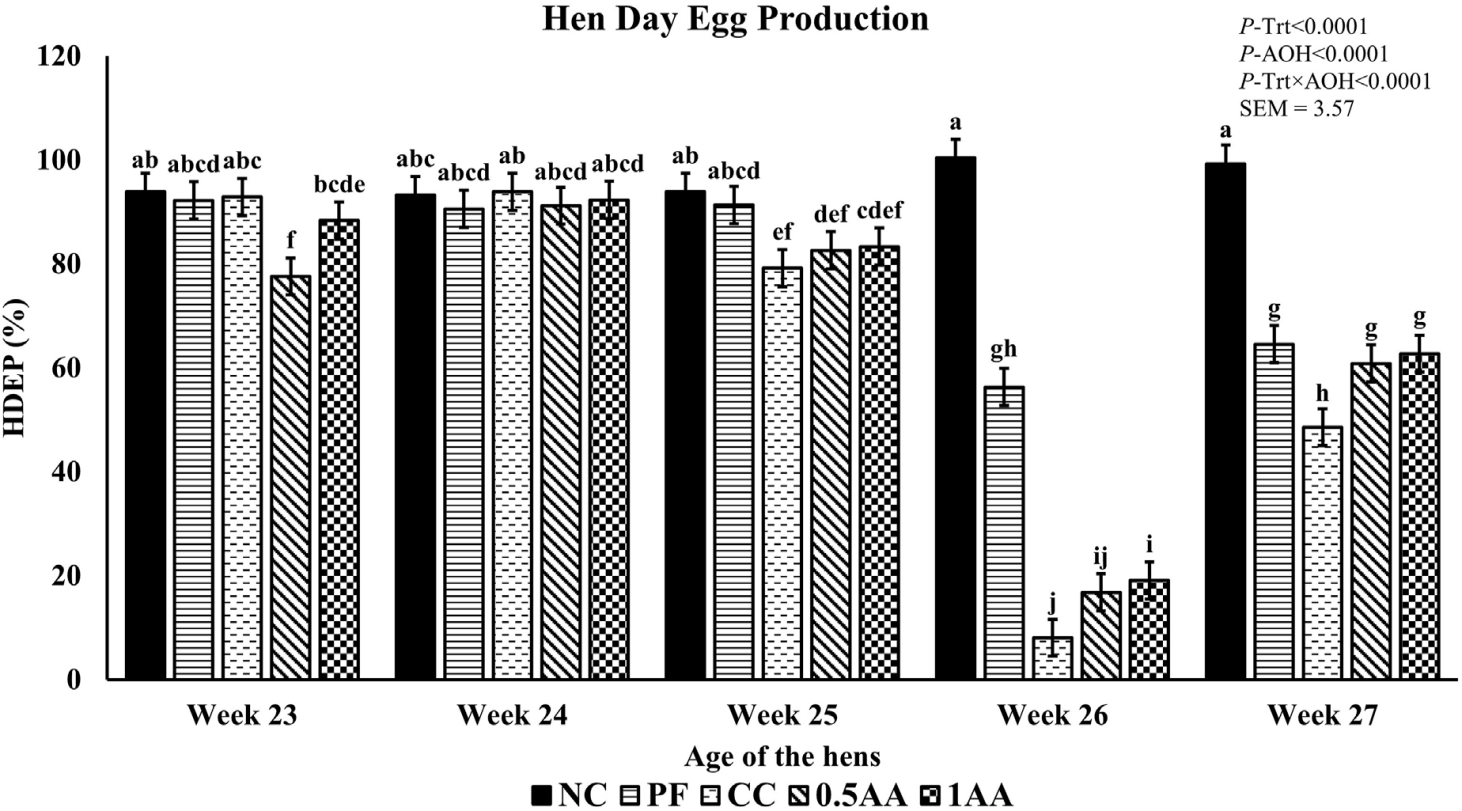
Effect of the inclusion of phytogenic feed additive *Artemisia annua* in the diets of laying hens on their weekly hen–day egg production from 23 weeks to 27 weeks of age. At 25 weeks of age, 12,500 *Eimeria maxima*, 12,500 *Eimeria tenella*, and 62,500 *Eimeria acervulina* oocysts were inoculated in laying hens grouped into challenged control, 0.5% *A. annua*, and 1% *A. annua*. NC: non-challenged control; PF: pair-fed control; CC: challenged control (12,500 *E. maxima*; 12,500 *E. tenella*; and 62,500 *E. acervulina* oocysts per mL); 0.5AA: challenged control with the inclusion of 0.5% *A. annua* in the diet; 1AA: challenged control with the inclusion of 1% *A. annua* in the diet. Before the *Eimeria* challenge at 25 weeks of age, treatments of NC, PF, and CC hens at 23 and 24 weeks of age were not different among themselves. Pair-feeding was carried out from 0–14 dpi after the *Eimeria* challenge. HDEP: hen–day egg production (%); Trt: treatment groups; AOH: age of the hens. Graphs not sharing the same superscripts (a–j) are significantly different at *p* < 0.05.

**FIGURE 3:**
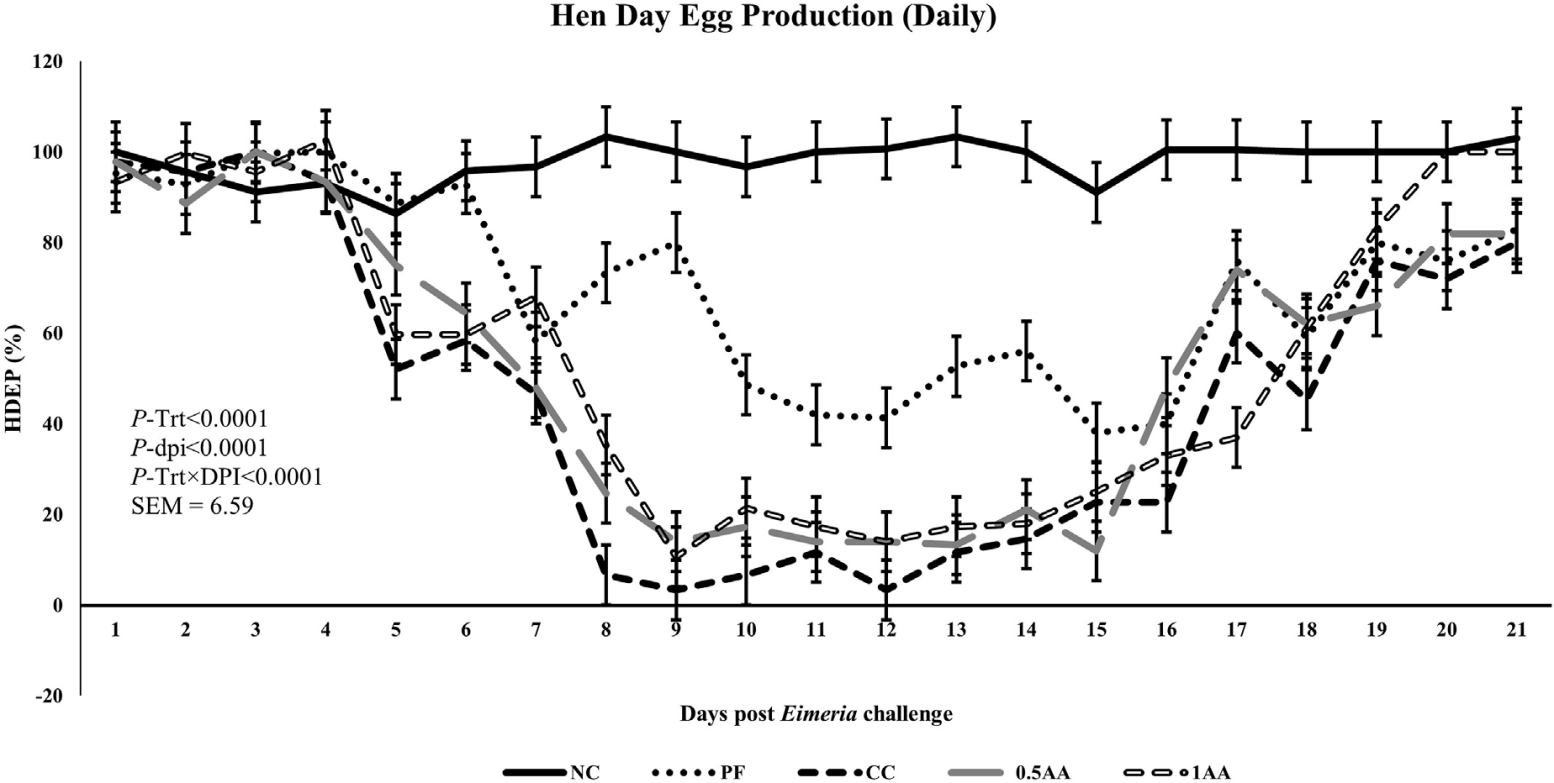
Effect of the inclusion of phytogenic feed additive *A. annua* in the diets of laying hens on their daily hen–day egg production from the day of a challenge until 21 days post-*Eimeria* challenge. At 25 weeks of age, 12,500 *E. maxima*, 12,500 *E. tenella*, and 62,500 *E. acervulina* oocysts were inoculated in laying hens grouped into challenged control, 0.5% *A. annua*, and 1% *A. annua*. NC: non-challenged control; PF: pair-fed control; CC: challenged control (12,500 *E. maxima*; 12,500 *E. tenella*; and 62,500 *E. acervulina* oocysts per mL); 0.5AA: challenged control with the inclusion of 0.5% *A. annua* in the diet; 1AA: challenged control with the inclusion of 1% *A. annua* in the diet. HDEP: hen–day egg production (%); Trt: treatment groups; dpi: days post-inoculation of *Eimeria* spp.

**FIGURE 4:**
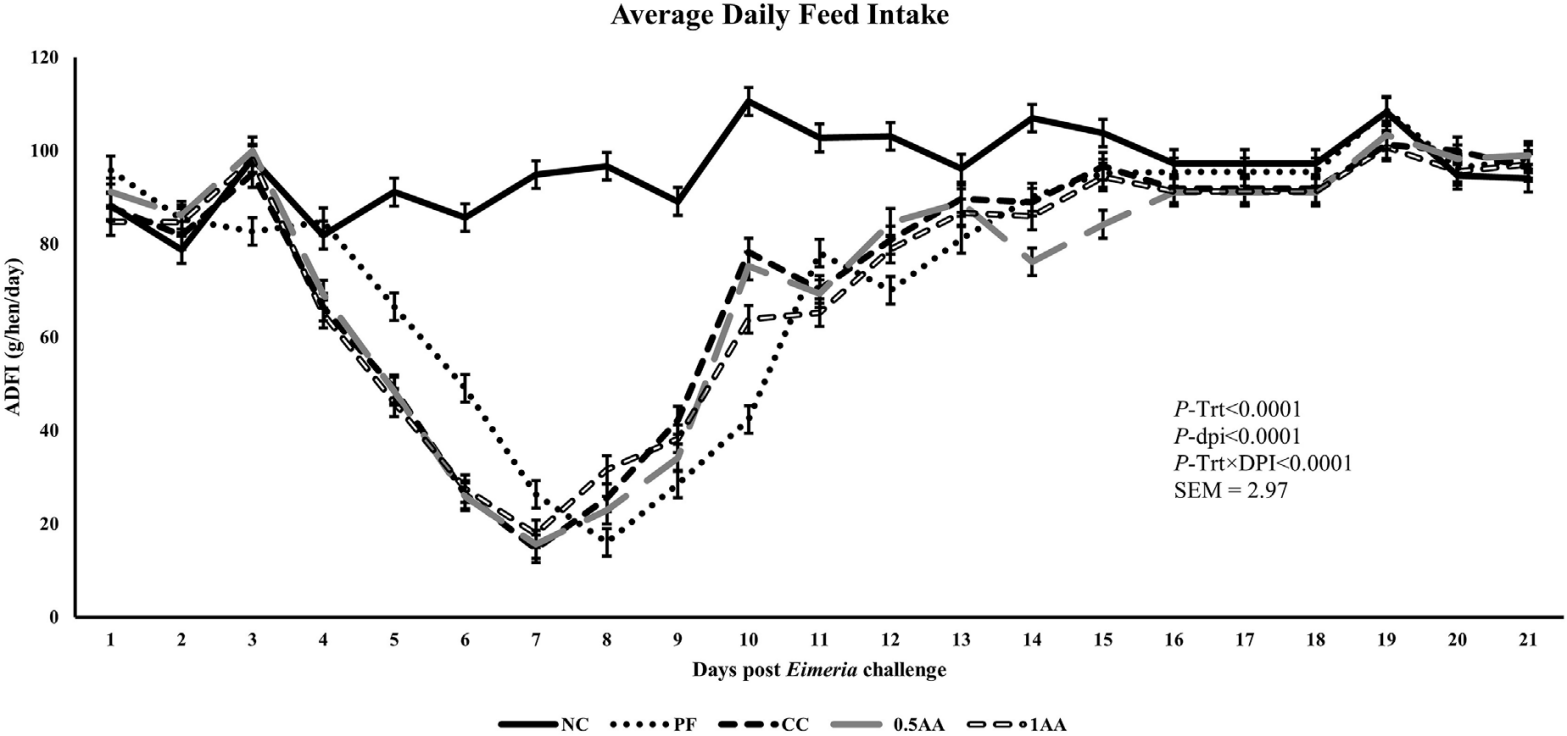
Effect of the inclusion of phytogenic feed additive *A. annua* in the diets of laying hens on their average daily feed intake from the day of a challenge until 21 days post-*Eimeria* challenge. At 25 weeks of age, 12,500 *E. maxima*, 12,500 *E. tenella*, and 62,500 *E. acervulina* oocysts were inoculated in laying hens grouped into challenged control, 0.5% *A. annua*, and 1% *A. annua*. NC: non-challenged control; PF: pair-fed control; CC: challenged control (12,500 *E. maxima*; 12,500 *E. tenella*; and 62,500 *E. acervulina* oocysts per mL); 0.5AA: challenged control with the inclusion of 0.5% *A. annua* in the diet; 1AA: challenged control with the inclusion of 1% *A. annua* in the diet. ADFI: average daily feed intake (g/hen/day); Trt: treatment groups; dpi: days post-inoculation of *Eimeria* spp.

**FIGURE 5:**
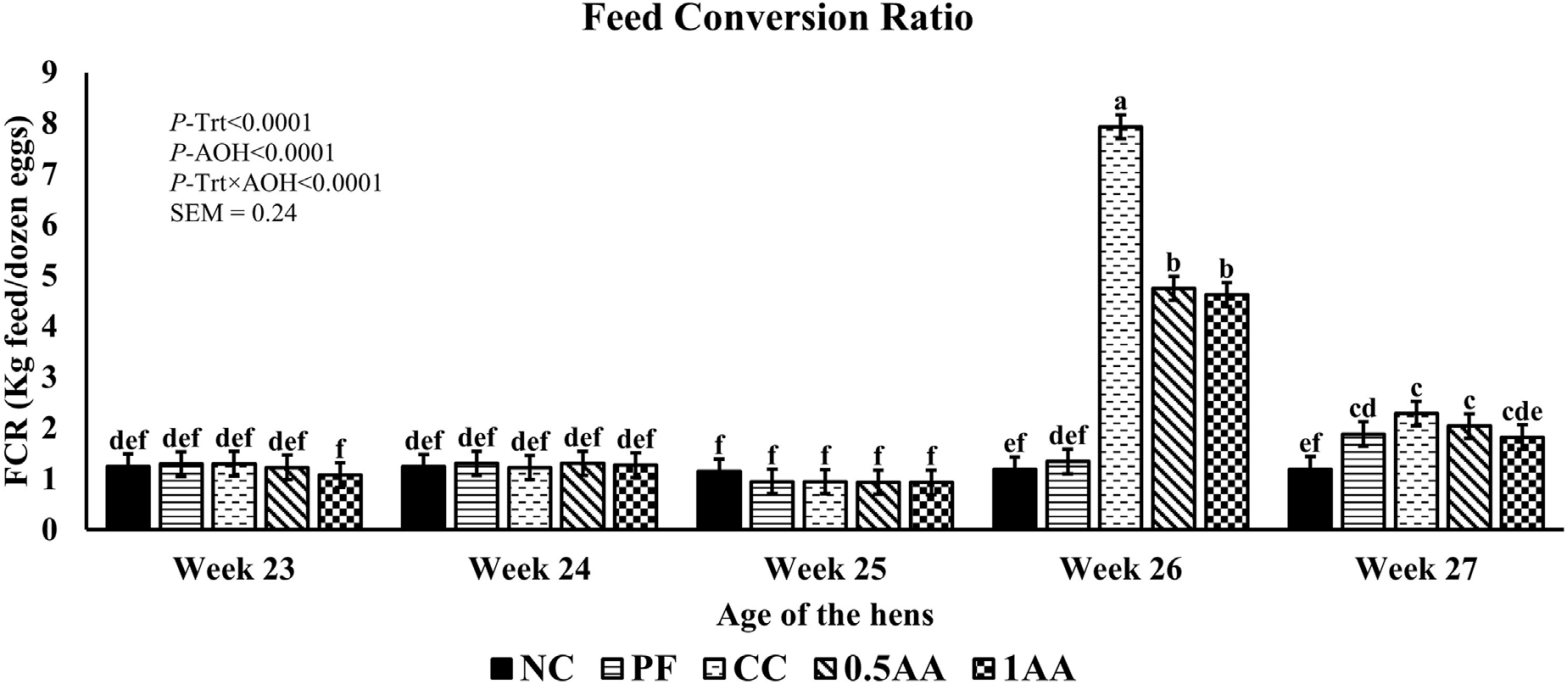
Effect of the inclusion of phytogenic feed additive *A. annua* in the diets of laying hens on their feed conversion ratio from 23 weeks to 27 weeks of age. At 25 weeks of age, 12,500 *E. maxima*, 12,500 *E. tenella*, and 62,500 *E. acervulina* oocysts were inoculated in laying hens grouped into challenged control, 0.5% *A. annua*, and 1% *A. annua*. NC: non-challenged control; PF: pair-fed control; CC: challenged control (12,500 *E. maxima*; 12,500 *E. tenella*; and 62,500 *E. acervulina* oocysts per mL); 0.5AA: challenged control with the inclusion of 0.5% *A. annua* in the diet; 1AA: challenged control with the inclusion of 1% *A. annua* in the diet. Before the *Eimeria* challenge at 25 weeks of age, treatments of NC, PF, and CC hens at 23 and 24 weeks of age were not different among themselves. Pair-feeding was carried out from 0–14 dpi after the *Eimeria* challenge. FCR: feed conversion ratio (kg feed per dozen eggs); Trt: treatment groups; AOH: age of the hens. Graphs not sharing the same superscripts (a–f) are significantly different at *p* < 0.05.

At 23 weeks of age, the HDEP of the laying hens fed AA was reduced, but it recovered back to the level of the NC group by 24 weeks of age (*p <* 0.0001; Figure 2). Following the *Eimeria* challenge at 25 weeks of age, the HDEP was significantly reduced in *Eimeria*-challenged hens compared to PF and NC hens. The daily egg production of laying hens started to decrease in *Eimeria*-challenged groups from 5 dpi, peaked at 9 dpi, and was not able to recover by 21 dpi, except for those hens fed 1% AA (Figure 3). The laying hens fed 0.5% and 1% AA increased their overall egg production (0–21 dpi) from 46.7% in CC hens to 52.9% and 54.8%, respectively. However, the HDEP was still lower than that of the PF (70.2%) and NC (98.0%) groups. The difference in the HDEP (0–21 dpi) between NC and PF hens was 27.8%, while it was observed to be 23.5% between PF and CC hens. The results for the ADFI following the *Eimeria* challenge at 25 weeks of age have a similar trend for the daily HDEP, as shown in Figure 4. An interaction was observed between different treatment groups and dpi for the ADFI (*p* < 0.0001). The ADFI started to decrease in challenged hens, beginning at 4 dpi, peaking at 7 dpi, and recovering to the same level as that of NC hens at 19 dpi. However, feeding AA did not have any beneficial effect on improving the ADFI. The FCR increased significantly in CC hens (7.93 kg feed/dozen of eggs), followed by 0.5AA (4.75 kg feed/dozen eggs) and 1AA (4.63 kg feed/dozen eggs) hens compared to NC hens (1.19 kg feed/dozen of eggs; *p <* 0.0001) at 26 weeks of age, as shown in Figure 5. However, 0.5% AA and 1%AA inclusion reduced the overall FCR by 0.50 and 0.61 during the challenged period (25–27 weeks of age), respectively.

### Gut permeability, lesion score, and oocyst shedding

**FIGURE 6:**
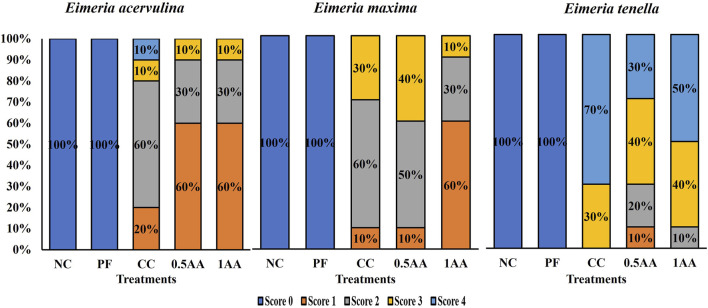
Effect of the inclusion of phytogenic feed additive *A. annua* in the diets of laying hens on their intestinal lesion score for *E. acervulina* (duodenum), *E. maxima* (part of the jejunum and ileum), and *E. tenella* (ceca) at 6 days post-*Eimeria* challenge. The lesion scores were ranked based on the severity of lesions from 0 to 4, with 0 indicating no lesions and 4 indicating extremely severe lesions. At 25 weeks of age, 12,500 *E. maxima*, 12,500 *E. tenella*, and 62,500 *E. acervulina* oocysts were inoculated in laying hens grouped into challenged control, 0.5% *A. annua*, and 1% *A. annua*. NC: non-challenged control; PF: pair-fed control; CC: challenged control (12,500 *E. maxima*; 12,500 *E. tenella*; and 62,500 *E. acervulina* oocysts per mL); 0.5AA: challenged control with the inclusion of 0.5% *A. annua* in the diet; 1AA: challenged control with the inclusion of 1% *A. annua* in the diet; dpi: days post-inoculation of *Eimeria* spp.

The *Eimeria*-challenged hens showed higher gut permeability than non-challenged hens (*p <* 0.0001; Table 5). Among the challenged hens, the inclusion of AA reduced the gut permeability by 0.14 μg/mL compared to CC hens (0.48 μg/mL). The inclusion of AA in the diet resulted in a reduction in the severity of intestinal lesions, transitioning from higher to lower lesion scores across all three sections of the intestine (Figure 6). Oocyst shedding was only observed in *Eimeria*-challenged hens, confirming that the NC and PF hens were not infected. The inclusion of 0.5% AA in the diet increased the oocyst shedding of *E. tenella* and reduced the oocyst shedding of *E. acervulina* (*p <* 0.05; Table 5).

**TABLE 5:** Effects of the dietary inclusion of phytogenic feed additive *Artemisia annua* in the diets of laying hens on their gut permeability at 5 dpi and oocyst shedding of *Eimeria acervulina, Eimeria maxima*, and *Eimeria tenella* from 5–8 dpi.

Items^a^	Gut permeability (µg/mL)	*E. acervulina* (Log_10_)	*E. maxima* (Log_10_)	*E. tenella* (Log_10_)
NC	0.01^b^	0.00^c^	0.00^b^	0.00^c^
PF	0.01^b^	0.00^c^	0.00^b^	0.00^c^
CC	0.48^a^	5.43^a^	3.90^a^	4.40^b^
0.5AA	0.34^a^	5.22^b^	4.02^a^	5.22^a^
1AA	0.34^a^	5.29^a^	3.84^a^	4.57^ab^
SEM	0.06	0.07	0.07	0.09
*p*-value	<0.0001	<0.0001	<0.0001	<0.0001

^a-c^values within columns not sharing the same superscripts are significantly different at *p* < 0.05.

^a^ NC, non-challenged control; PF, pair-fed control; CC, challenged control (12,500 *E. maxima*; 12,500 *E. tenella*; and 62,500 *E. acervulina* oocysts per mL); 0.5AA, challenged control with the inclusion of 0.5% *Artemisia annua* in the diet; 1AA, challenged control with the inclusion of 1% *A. annua* in the diet; dpi: days post-inoculation of *Eimeria* spp.

### Gene expression

The effects of feeding AA leaves on the intestinal barrier function and nutrient transporter gene expression of the laying hens post-*Eimeria* challenge are shown in Table 6. At 6 and 14 dpi, the expression of JAM-2, OCLN, ZO-2, and MUC-2 was downregulated in *Eimeria*-challenged hens compared to non-challenged hens, whereas the expression of CLDN-1 was upregulated only at 6 dpi (*p <* 0.05). The inclusion of AA leaves upregulated the mRNA expression of iNOS at 6, 14, and 21 dpi (*p <* 0.05). The expression of nutrient transporter genes (b^0+^ AT, EAAT-3, y^+^LAT, b^0^AT, pepT-1, SGLT-1, and GLUT-1) was slightly upregulated in the PF group, whereas they were downregulated in the *Eimeria*-challenged hens at 6 dpi (*p <* 0.05). At 14 dpi, 1% AA inclusion upregulated the amino acid transporter genes compared to CC hens but remained lower than that of the NC and PF hens (*p <* 0.05). Furthermore, y^+^LAT, GLUT-1, and SGLT-1 expression was downregulated in PF hens compared to NC hens (*p <* 0.05; Table 6). At 6 dpi, the expression of cytokines IL-10 and IFN-γ in *Eimeria*-challenged hens (CC and 1AA) was upregulated compared to non-challenged hens. However, this upregulation was lower in hens grouped in 0.5AA than that in CC and 1AA hens (*p <* 0.05; Table 7). On the other hand, the expression of IL-4 was downregulated by the inclusion of AA (*p =* 0.0808). No significant differences in any genes among the treatment groups were observed at 14 dpi (*p* > 0.05). However, at 21 dpi, all treatments exhibited lower INF-γ expression than the NC treatment (*p* < 0.05).

**TABLE 6:** Effects of the dietary inclusion of phytogenic feed additive *Artemisia annua* in the diet of laying hens on their intestinal barrier function and nutrient transporter genes 6, 14, and 21 dpi.

	6 dpi												
	CLDN-1	JAM-2	OCLN	ZO-2	iNOS	MUC-2	b^0+,^ AT	EAAT-3	y^+^LAT	pepT-1	b^0^ AT	GLUT-1	SGLT-1
NC	1.00^b^	1.00^ab^	1.00^a^	1.00	1.00^c^	1.00^a^	1.00^b^	1.00^a^	1.00^a^	1.00^ab^	1.00^a^	1.00^a^	1.00^a^
PF	1.27^b^	1.24^a^	1.18^a^	1.09	1.26^c^	1.08^a^	1.30^a^	1.08^a^	1.12^a^	1.18^a^	1.44^a^	1.09^a^	1.20^a^
CC	10.33^a^	0.72^c^	0.47^b^	0.85	3.23^bc^	0.40^b^	0.21^c^	0.38^b^	0.10^b^	0.62^bc^	0.39^b^	0.17^b^	0.35^b^
0.5AA	10.76^a^	0.80^bc^	0.51^b^	0.87	6.66^a^	0.36^b^	0.11^c^	0.31^b^	0.04^b^	0.34^c^	0.41^b^	0.04^b^	0.25^b^
1AA	11.05^a^	0.78^bc^	0.50^b^	0.80	4.56^ab^	0.32^b^	0.20^c^	0.36^b^	0.06^b^	0.60^bc^	0.25^b^	0.17^b^	0.32^b^
SEM	1.22	0.09	0.08	0.11	0.93	0.08	0.08	0.06	0.06	0.16	0.19	0.13	0.09
*p*-value	<0.0001	0.0042	<0.0001	0.3534	0.0018	<0.0001	<0.0001	<0.0001	<0.0001	0.0133	0.0008	<0.0001	<0.0001
14 dpi													
NC	1.00^b^	1.00	1.00^a^	1.00^a^	1.00^a^	1.00^ab^	1.00^ab^	1.00^a^	1.00^a^	1.00^a^	1.00	1.00^a^	1.00^a^
PF	1.55^a^	1.04	0.78^a^	1.02^a^	0.93^a^	1.15^a^	1.41^a^	1.03^a^	0.60^b^	0.90^a^	1.39	0.59^b^	0.66^b^
CC	0.48^cd^	0.72	0.39^b^	0.54^b^	0.55^c^	0.58^b^	0.50^b^	0.46^b^	0.38^b^	0.45^b^	0.29	0.42^b^	0.36^c^
0.5AA	0.44^d^	0.71	0.36^b^	0.50^b^	0.68^bc^	0.53^b^	0.48^b^	0.38^b^	0.33^b^	0.22^b^	0.2	0.23^b^	0.34^c^
1AA	0.77^bc^	1.00	0.50^b^	0.69^b^	0.85^ab^	0.78^ab^	0.84^ab^	0.71^ab^	0.46^b^	0.36^b^	0.58	0.36^b^	0.57^b^
SEM	0.10	0.13	0.08	0.09	0.07	0.17	0.23	0.14	0.11	0.40	0.33	0.12	0.06
*p*-value	<0.0001	0.2022	<0.0001	0.0004	0.0019	0.0717	0.0538	0.0089	0.0029	0.0018	0.1068	0.0026	<0.0001
21 dpi													
NC	1.00	1.00^b^	1.00	1.00	1.00^c^	1.00	1.00	1.00	1.00	1.00	1.00	1.00	1.00
PF	0.97	1.07^b^	1.11	1.13	1.30^ab^	1.23	0.95	1.24	0.99	1.18	1.14	0.96	1.23
CC	1.25	1.32^a^	1.28	1.20	1.28^bc^	0.91	0.99	1.21	1.17	1.18	1.29	1.13	1.38
0.5AA	1.36	1.40^a^	1.38	1.08	1.68^a^	1.16	1.27	1.11	0.73	0.88	1.26	0.65	0.95
1AA	1.00	1.37^a^	1.23	1.04	1.38^ab^	1.03	1.03	1.22	1.10	0.83	1.18	1.00	1.14
SEM	0.22	0.07	0.10	0.09	0.12	0.10	0.09	0.09	0.18	0.16	0.17	0.21	0.12
*p*-value	0.656	0.001	0.1158	0.5379	0.0113	0.1695	0.1165	0.3472	0.508	0.387	0.7867	0.5943	0.124

^a-c^values within columns not sharing the same superscripts are significantly different at *p* < 0.05.

^a^ NC: non-challenged control; PF: pair-fed control; CC: challenged control (12,500 *E. maxima*; 12,500 *E. tenella*; and 62,500 *E. acervulina* oocysts per mL); 0.5AA: challenged control with the inclusion of 0.5% *Artemisia annua* in the diet; 1AA: challenged control with the inclusion of 1% *A. annua* in the diet.

CLDN-1, claudin-1; ZO-2, zonula occludin-2; OCLN, occludin; JAM-2, junctional adhesion molecule-2; MUC-2, mucin-2; iNOS, inducible nitric oxide synthase; b^0,+^ AT, solute carrier family 7, member 9; EAAT-3, excitatory amino acid transporter 3; pepT-1; b^0^ AT, solute carrier family 6, member 19; y^+^ LAT-1, y^+^ L amino acid transporter-1; SGLT-1, sodium-glucose transporter-1; GLUT-1, glucose transporter-1; dpi, days post-inoculation of *Eimeria* spp.

**TABLE 7:** Effects of the dietary inclusion of phytogenic feed additive *Artemisia annua* in the diets of laying hens on their expression of inflammatory cytokines at 6, 14, and 21 dpi.

	6 dpi					
Items	IL-1β	IL-4	IL-10	IL-17	IFN-γ	TNF-α
NC	1.00	1.00^ab^	1.00^b^	1.00	1.00^c^	1.00^a^
PF	0.96	1.22^a^	1.15^b^	0.56	1.36^c^	1.09^a^
CC	1.35	1.14^ab^	4.07^a^	0.86	10.40^a^	0.67^b^
0.5AA	1.41	0.53^b^	1.69^b^	0.92	5.70^b^	0.62^b^
1AA	1.30	0.50^b^	4.01^a^	0.81	10.04^a^	0.59^b^
SEM	0.23	0.22	0.59	0.26	1.29	0.09
*p*-value	0.5231	0.0808	0.0016	0.8000	<0.0001	0.0021
14 dpi						
NC	1.00	1.00	1.00	1.00	1.00	1.00
PF	1.23	0.98	0.82	1.41	0.74	1.16
CC	1.43	0.84	0.8	2.54	0.84	1.14
0.5AA	1.31	1.41	0.94	1.20	1.33	1.13
1AA	1.51	0.85	0.95	1.39	1.18	0.97
SEM	0.26	0.22	0.19	0.57	0.35	0.11
*p*-value	0.6935	0.3784	0.9338	0.3757	0.7434	0.6847
21 dpi						
NC	1.00	1.00	1.00	1.00^a^	1.00^a^	1.00
PF	0.72	1.35	0.59	0.34^b^	0.35^b^	0.83
CC	0.91	1.04	0.91	0.52^b^	0.45^b^	0.92
0.5AA	0.99	0.83	0.77	0.68^ab^	0.47^b^	0.83
1AA	1.15	1.00	0.80	0.65^ab^	0.33^b^	0.99
SEM	0.14	0.18	0.16	0.15	0.11	0.09
*p*-value	0.3253	0.3497	0.4867	0.0642	0.0031	0.6405

^a-c^values within columns not sharing the same superscripts are significantly different at *p* < 0.05.

^a^ NC, non-challenged control; PF, pair-fed control; CC, challenged control (12,500 *E. maxima*; 12,500 *E. tenella*; and 62,500 *E. acervulina* oocysts per mL); 0.5AA, challenged control with the inclusion of 0.5% *Artemisia annua* in the diet; 1AA, challenged control with the inclusion of 1% *A. annua* in the diet.

IL-1β, interleukin-1 β; IL-4, interleukin-4; IL-10, interleukin-10; IL-17, interleukin-17 A; TNF-α, tumor necrosis factor-α; IFN-γ, interferon-γ; dpi, days post-inoculation of *Eimeria* spp.

### Small intestine histomorphology

The effect of dietary inclusion of AA leaves in a laying hen diet infected with *Eimeria* spp. is presented in Table 8. At 6 dpi, the VH of the duodenum and jejunum decreased, whereas CD increased by the *Eimeria* challenge, resulting in a lower VH-to-CD ratio (*p <* 0.05). Although no significant difference was observed in ileal VH, CD was significantly increased, lowering the VH-to-CD ratio in challenged hens (*p <* 0.05). The inclusion of 0.5% AA resulted in a higher VH in all three sections relative to CC at 6 dpi. At 14 and 21 dpi, the inclusion of AA reduced the CD in challenged hens in all three sections of the intestine compared to the CC hens (*p <* 0.1). However, VH of the jejunum increased at 6 dpi (*p* < 0.05), and as feed restriction progressed in the PF group, a slight reduction in jejunum VH was observed at 14 and 21 dpi (*p* > 0.05). Although a higher jejunum CD was observed in challenged hens at 14 dpi (*p* < 0.05), it became similar to that of NC hens by 21 dpi. In the ileum, the VH decreased and CD increased through 21 dpi in the PF group compared to the NC group (*p* < 0.05).

**TABLE 8:** Effects of the dietary inclusion of phytogenic feed additive *Artemisia annua* in the diets of laying hens on their small intestinal histomorphology at 6, 14, and 21 dpi.

	Duodenum								
	6 dpi			14 dpi			21 dpi		
	VH	CD	VHCD	VH	CD	VHCD	VH	CD	VHCD
NC	1,827.0^a^	121.4^b^	15.78^a^	1,752.7	121.8^d^	14.46^a^	1,845.1	130.1^c^	14.43
PF	1,560.8^b^	145.9^b^	11.14^b^	1,790.6	159.2^d^	11.32^b^	1,730.9	142.1^bc^	12.50
CC	1,110.8^c^	448.8^a^	2.55^c^	1,620.8	379.4^a^	4.33^d^	1,900	186.1^a^	10.86
0.5AA	1,233.1^c^	383.0^a^	3.32^c^	1,775.0	299.8^b^	6.15^cd^	1,886.4	176.3^ab^	10.91
1AA	1,229.9^c^	406.1^a^	3.09^c^	1,753.0	233.8^c^	7.71^c^	1,692.3	151.1^abc^	11.43
SEM	62.01	26.75	0.93	76.52	17.42	0.63	65.12	14.71	1.02
*p*-value	<0.0001	<0.0001	<0.0001	0.5501	<0.0001	<0.0001	0.1211	0.0714	0.1116
Jejunum									
NC	1,100.2^ab^	97.1^c^	11.35^a^	1,294.2	113.6^c^	11.70^a^	1,056.5	110.0	9.88
PF	1,277.5^a^	133.5^c^	9.66^b^	1,152.1	144.4^b^	8.09^b^	938.0	109.5	9.21
CC	932.3^b^	416.1^a^	2.29^d^	1,157.5	183.4^a^	6.43^b^	1,084.7	135.1	8.36
0.5AA	966.5^b^	397.3^b^	3.44^c^	1,198.2	183.2^a^	6.61^b^	1,054.1	143.0	7.70
1AA	912.5^b^	351.2^ab^	2.70^cd^	1,117.1	149.5^b^	7.63^b^	1,001.9	121.0	8.29
SEM	80.16	28.34	0.38	60.15	10.35	0.68	47.99	13.97	0.78
*p*-value	0.0222	<0.0001	<0.0001	0.3105	0.0005	0.0001	0.2505	0.362	0.3359
Ileum									
NC	686.1	84.4^c^	8.23^a^	760.4^a^	81.5^c^	9.68^a^	759.2^ab^	79.2^c^	8.79
PF	707.3	107.0^c^	6.78^b^	616.8^b^	96.8^bc^	6.61^b^	607.1^b^	86.7^bc^	8.04
CC	739.5	335.4^a^	2.26^c^	807.1^a^	133.0^a^	6.33^b^	864.5^a^	122.3^a^	7.13
0.5AA	772.1	356.7^b^	3.04^c^	794.8^a^	111.7^ab^	7.15^b^	784.1^a^	113.7^ab^	7.22
1AA	694.3	246.5^b^	2.88^c^	869.5^a^	126.7^a^	6.34^b^	859.3^a^	96.0^abc^	9.10
SEM	41.15	19.88	0.34	31.0	9.83	0.71	53.1	10.38	0.69
*p*-value	0.6523	<0.0001	<0.0001	0.0022	0.0082	0.0164	0.0173	0.0414	0.1972

^a-c^values within columns not sharing the same superscripts are significantly different at *p* < 0.05.

^a^ NC, non-challenged control; PF, pair-fed control; CC, challenged control (12,500 *E. maxima*; 12,500 *E. tenella*; and 62,500 *E. acervulina* oocysts per mL); 0.5AA, challenged control with the inclusion of 0.5% *Artemisia annua* in the diet; 1AA, challenged control with the inclusion of 1% *A. annua* in the diet.

VH, villus height (µm); CD, crypt depth (µm); VHCD, villus height-to-crypt depth ratio; dpi, days post-inoculation of *Eimeria* spp.

### Flow cytometry

**FIGURE 7:**
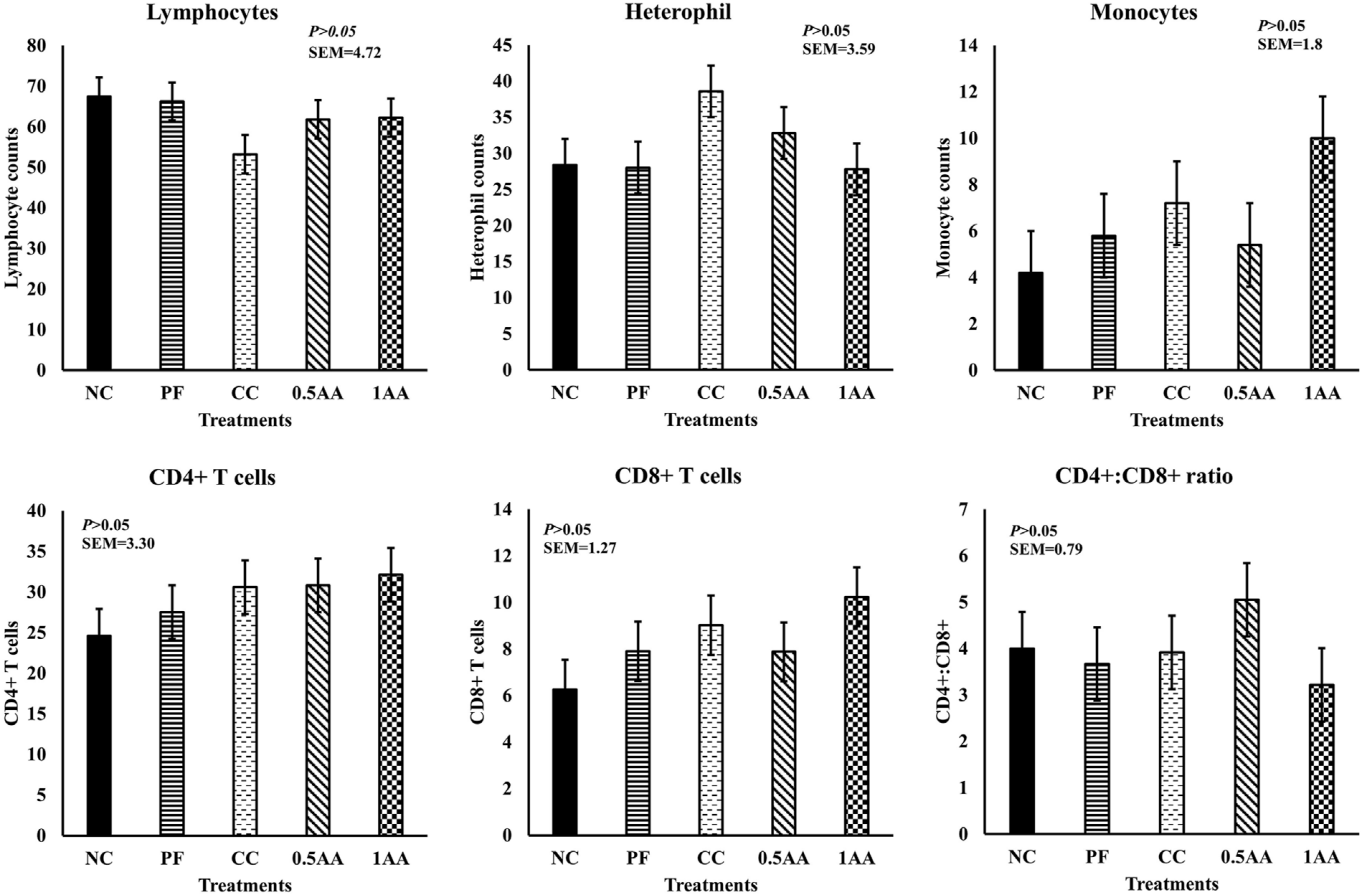
Effect of the inclusion of phytogenic feed additive *A. annua* in the diets of laying hens on their peripheral mononuclear blood cell count and CD4^+^ and CD8^+^ T cell population 14 days post-*Eimeria* challenge. At 25 weeks of age, 12,500 *E. maxima*, 12,500 *E. maxima*, and 62,500 *E. tenella* oocysts were inoculated in laying hens grouped into challenged control, 0.5% *A. annua*, and 1% *A. annua*. NC: non-challenged control; PF: pair-fed control; CC: challenged control (12,500 *E. maxima*; 12,500 *E. tenella*; and 62,500 *E. acervulina* oocysts per mL); 0.5AA: challenged control with the inclusion of 0.5% *A. annua* in the diet; 1AA: challenged control with the inclusion of 1% *A. annua* in the diet; dpi: days post-inoculation of *Eimeria* spp.

No significant differences were observed in the peripheral mononuclear blood cell count at 14 dpi (*p >* 0.05; Figure 7). However, the monocyte counts in the peripheral blood increased by 71% in hens grouped in CC and by 138% in those fed 1% AA compared to NC hens. Likewise, no significant differences were observed for CD4^+^, CD8^+^, and their ratio.

### Oxidative status

**FIGURE 8:**
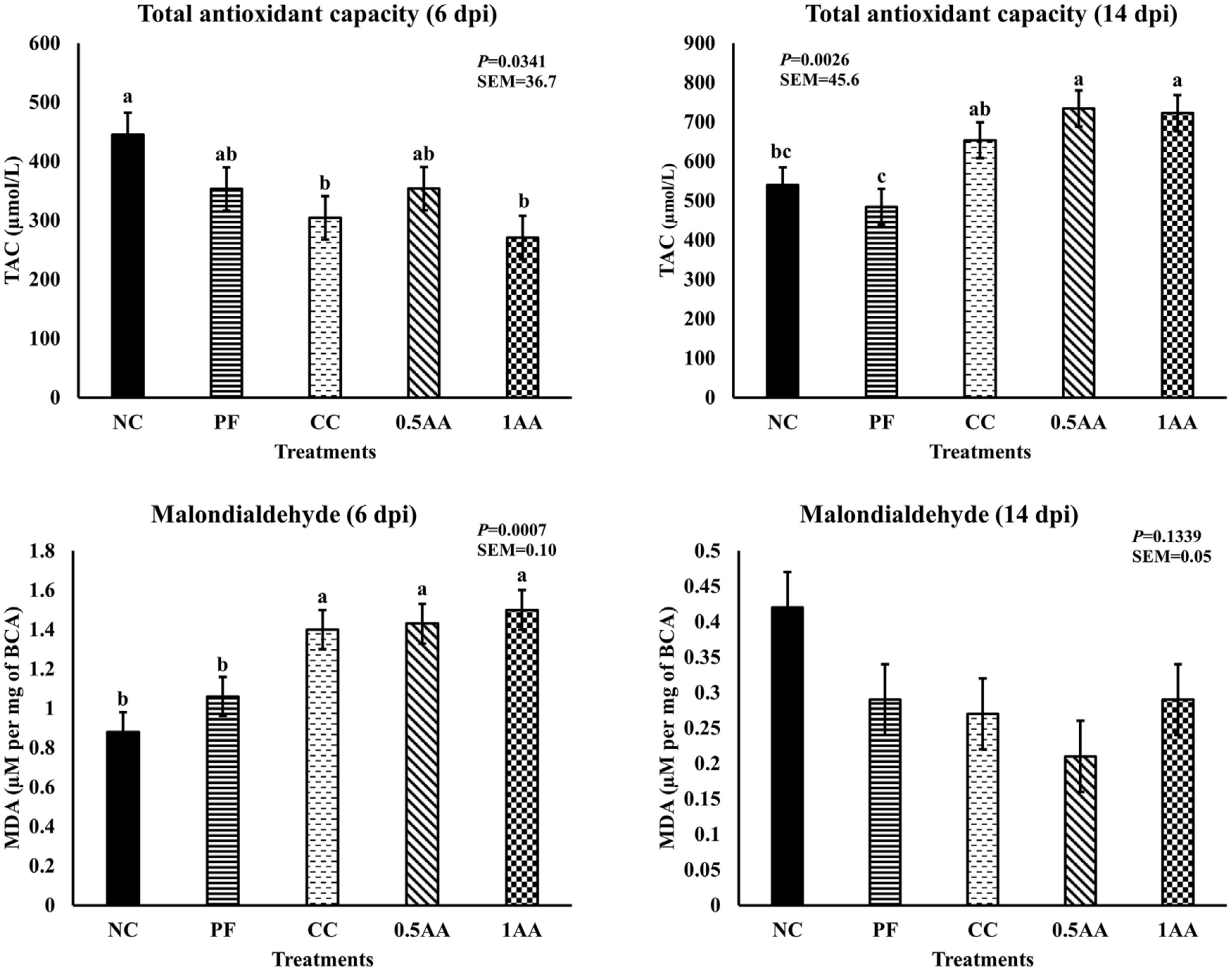
Effect of the inclusion of phytogenic feed additive *A. annua* in the diets of laying hens on their oxidative status at 6 and 14days post-*Eimeria* challenge. At 25 weeks of age, 12,500 *E. maxima*, 12,500 *E. maxima*, and 62,500 *E. tenella* oocysts were inoculated in laying hens grouped into challenged control, 0.5% *A. annua*, and 1% *A. annua*. NC: non-challenged control; PF: pair-fed control; CC: challenged control (12,500 *E. maxima*; 12,500 *E. tenella*; and 62,500 *E. acervulina* oocysts per mL); 0.5AA: challenged control with the inclusion of 0.5% *A. annua* in the diet; 1AA: challenged control with the inclusion of 1% *A. annua* in the diet; TAC: total antioxidant capacity (µmol/L); MDA: malondialdehyde (µM per mg of BCA); dpi: days post-inoculation of *Eimeria* spp. Graphs not sharing the same superscripts (a–b) are significantly different at *p* < 0.05.

The effect of the inclusion of AA leaves on the oxidative status of laying hens post-*Eimeria* challenge is shown in Figure 8. At 6 dpi, *Eimeria* challenges and pair feeding reduced the total antioxidant capacity; however, at 14 dpi, the TAC increased in challenged hens but not in the PF group (*p <* 0.05). Likewise, MDA levels increased in *Eimeria*-challenged hens at 6 dpi (*p* < 0.05) and became similar to that of NC hens by 14 dpi. The inclusion of AA did not have a beneficial effect on improving the oxidative status of the laying hens post-*Eimeria* challenge.

## Discussion

In this study, we investigated the role of phytogenic feed additive (*A. annua*) supplementation on the performance, intestinal barrier functions, oxidative status, and immune response of laying hens infected with mixed *Eimeria* species. Supplementing the phytogenic feed additive (*A. annua*) showed some beneficial effects on improving the laying hen performance, intestinal barrier functions, and, to some extent, immune responses against *Eimeria* spp. tested in the current study.

As expected, *Eimeria* infection reduced the egg-laying performance and feed intake of laying hens while increasing the feed conversion ratio over the 21-day period. Although the inclusion of AA did not improve the growth performance, it did improve the egg production and feed efficiency of the laying hens. The results of growth performance were different from those observed previously, where the inclusion of the AA leaves or its extract (artemisinin) at various levels prevented weight loss in broilers compared to *Eimeria-*challenged hens (Allen et al., 1997; Hady and Zaki, 2012; Pop et al., 2015). However, in this study, AA inclusion was ineffective in preventing BW loss. As the hens were not growing, they might have utilized their fat reserve to lay eggs during the acute phase (0–6 dpi) while simultaneously fighting the infection (Sharma et al., 2024b). In the current study, the distinct smell and bitter taste of AA led to strong feed refusal in the lower AA-inclusion group compared to the higher inclusion group. However, the exact reason for strong feed refusal in the lower inclusion group remains unclear. Consequently, this resulted in a decrease in body weight and egg production in the 0.5% AA-inclusion group during the adaptation period (Engberg et al., 2012). The literature on the effect of AA on laying hen performance is scarce; however, the increase in egg production in hens during the challenge period might be due to the antioxidant, antimicrobial, and antiprotozoal properties of artemisinin (Allen et al., 1997; Del Cacho et al., 2010; Engberg et al., 2012; Baghban-Kanani et al., 2019). The difference between NC and CC hens (51.3%) for the HDEP was attributed to the reduced feed intake and symptoms associated with disease during the infection period. Additionally, the difference in the HDEP between PF and CC hens (23.5%) might be attributed to the nutritional redistribution associated with inflammation and immune responses, whereas 27.8% was associated with reduced feed intake following coccidiosis.

Gastrointestinal health is of great importance in poultry as it affects nutrient digestion, absorption, and utilization, and, most importantly, if compromised, affects the health and welfare of the chickens (Choi et al., 2023a; Tompkins et al., 2023; Sharma et al., 2024a; 2024b). The findings from the current study confirmed that *Eimeria* species damage the intestinal tract with mild-to-severe intestinal lesions and structural damage to the intestinal architecture of the villi, ultimately affecting the performance of the laying hens. However, the inclusion of the AA in the diet reduced the severity of intestinal lesions in the duodenum, jejunum, ileum, and ceca. Previously, it had been observed that either the AA leaves or its extract (artemisinin) reduced the lesion score, oocyst shedding, and symptoms related to coccidiosis (Allen et al., 1997; Almeida et al., 2012; Hady and Zaki, 2012; Kaboutari et al., 2014; Mo et al., 2014; Jiao et al., 2018). Unlike the findings obtained by Allen et al. (1997), who reported a significant reduction in the oocyst shedding of *E. tenella*, our study observed only a slight decrease in the oocyst shedding of *E. acervulina*. The reduced oocyst shedding observed may be attributed to the artemisinin derived from the AA leaves, which alters the formation of the oocyst wall, resulting in both death and reduced sporulation rate of oocysts (Del Cacho et al., 2010; Fatemi et al., 2015; Pop et al., 2015). The variation in oocyst shedding observed between the studies might be due to differences in the source of artemisinin used, such as extracted artemisinin or artemisinin released from the AA leaves. In this study, the differences observed in *E. tenella* and *E. acervulina* oocyst shedding between the 0.5% and 1% AA inclusion groups might be associated with the environments where they reside. Unlike *E. acervulina*, *E. tenella* develops under relatively anaerobic conditions in the ceca, filled with cecal contents. This anaerobic environment in the ceca may require a higher concentration of artemisinin to exert its effects (Allen et al., 1997). Furthermore, it was reported that artemisinin from AA leaves induces apoptotic events in *Eimeria*-infected cells, resulting in increased apoptosis of merozoites and protozoal host cells. This ultimately reduces the second-generation merozoites released into the intestinal lumen (Del Cacho et al., 2010; Mo et al., 2014). Consequently, this reduces the intestinal lesion score and gut permeability in the infected hosts, as observed in this study.

In this study, the oxidative status of the laying hens following the *Eimeria* challenge among the infected groups was not different. It has been observed that artemisinin is capable of inducing oxidative stress by increasing free radicals in specific host cells where *Eimeria* resides. This increased production of free radicals is suggested to kill the intracellular parasite, in this case, *Eimeria* (Meshnick et al., 1989; Kaboutari et al., 2014; Jiao et al., 2018). Moreover, the inducible nitric oxide synthase (iNOS) expression was upregulated in hens in 0.5% AA and 1% AA groups compared to CC hens. Inducible nitric oxide synthase helps in the production of nitric oxide, which induces oxidative stress, thereby limiting *Eimeria* multiplication in the host cells (Castro et al., 2020). Although artemisinin has an antioxidant capacity to prevent free radicals and lipid peroxidation in non-infected birds (Drǎgan et al., 2014), the *Eimeria* infection may have changed its functions to specifically increase oxidative stress in infected host cells to cause apoptosis of the infected host cells and merozoites residing in them (Meshnick et al., 1989; Kaboutari et al., 2014; Jiao et al., 2018).

As previously observed, *Eimeria* infection significantly influenced duodenal, jejunal, and ileal morphology by decreasing the VH and increasing the CD of laying hens (Sharma et al., 2024a; 2024b). Conversely, the dietary inclusion of AA partially alleviated the negative effect of coccidiosis on the small intestinal morphometric architecture during the recovery phase (8–21 dpi). The selective apoptosis of *Eimeria*-infected host cells by artemisinin might have facilitated the quick recovery of intestinal villi (Mo et al., 2014; Jiao et al., 2018). As observed in this study, the beneficial effect of AA on intestinal villus recovery may depend on multiple factors, including intestinal location, dpi, and dosage. For instance, 0.5% AA inclusion increased the VH in duodenal and jejunal regions, while 1% AA reduced the CD. However, 1% AA increased the VH and reduced the CD at 21 dpi in the ileum. In PF hens, a dietary restriction similar to that of CC hens from 0–14 dpi might be one of the reasons for the regression in intestinal villus height and increased crypt depth at 21 dpi. The regression in the villi may contribute to the downregulation of glucose transporters (GLUT-1 and SGLT-1) and y^+^LAT amino acid transporters at 14 dpi in the PF hens. This regression in villi and downregulation of specific nutrient transporters might have also played a role in the prominent production loss observed in PF groups by 14 dpi.

The breach in the intestinal mucosa by *Eimeria* triggers several signaling cascades, leading to the production of cytokines involved in inflammation (Lillehoj and Lillehoj, 2000; Yun et al., 2000; Hong et al., 2006a). Following the *Eimeria* infection, the expression of pro-inflammatory (IFN-γ) and anti-inflammatory cytokine (IL-10) increased in challenged birds, consistent with the findings from previous studies (Hong et al., 2006b; Sharma et al., 2024a; 2024b). However, 0.5% AA inclusion reduced the expression of both IFN-γ and IL-10 compared to the CC, suggesting that AA can reduce the inflammatory response of chickens by various means and help the normal functioning and upregulation of humoral responses (Song et al., 2017; Yang et al., 2021). Artemisinin is known to suppress the production of these cytokines by inhibiting the activation of NF-κB; however, the reason why the 1% inclusion of AA did not have the same effect as 0.5% AA inclusion remains unclear (Jiao et al., 2018). Furthermore, the inclusion of 1% AA upregulated cell-mediated immunity by increasing the monocyte counts and CD8^+^ cells, which are responsible for clearing intracellular pathogens (Liu et al., 2023; Shah et al., 2023). The partial reduction in inflammation and increased cell-mediated immunity to kill the intracellular *Eimeria* parasite, along with reduced intestinal lesions and permeability in AA-fed hens, might contribute to reducing energy allocation for host defense. This could potentially improve the performance of the hens by increasing the available energy for production, consequently increasing the HDEP by at least 6.25% in the AA groups.

## Conclusion

Based on the findings from the current study, it can be concluded that the adverse effects of the *Eimeria* challenge are associated with reduced feed intake and redistribution of the nutritional reserve of the host for inflammation and oxidative stress rather than the performance. However, dietary supplementation of the phytogenic feed additive (*A. annua* leaves) partially helped restore the egg production and intestinal integrity of the laying hens infected with *Eimeria* spp. Furthermore, it also helped modulate the immune response to alleviate the negative effect of coccidiosis on the performance. However, further studies need to be conducted to find the optimal dose of AA leaves to prevent feed refusal and improve the performance of laying hens.

## Data Availability

The raw data supporting the conclusion of this article will be made available by the authors, without undue reservation.
